# Bellevue Hospital Medical College and the Code of the Fathers

**Published:** 1884-02

**Authors:** 


### Correspondence.

### Bellevue Hospital Medical College and the Code of the Fathers.

(Letter from New York.)

Among the many curious phases which the code question has assumed during the past year's struggle in New York City, perhaps the most singular have been the vagaries pursued by the Bellevue Hospital Medical College, the standard-bearer of the Code of Ethics of the American Medical Association. No medical man who had watched the course of this school could repress a feeling of joy and pride that at least one college in the sink of iniquity, ycleped New York, had resisted the action of the anti-code poison which has permeated the profession of this city, and stood out bright and pure, a very beacon light in the midst of the ethical darkness which enshrouds the profession of medicine in New York State. But, alas! that we should be obliged to pull down the idol which our hands had reared; to tear down the bright but delusive image from the shrine in which pious and reverent hands had placed it. Alas! Bellevue is infected and in a more virulent form than the rest of the brethren. It is legalizing quacks by endorsing them, and here are the facts:

The first instance was the case of F. Eliseo Marini, who falsely claimed to have a diploma from the University of Naples, and in whom the Medical Society of the County of New York took a profound and almost paternal interest. So strong did this feeling become that the society arrested the said Marini in order to show him the error of his ways. First, in having an illegal diploma, and next, in not having it endorsed as required by law. Between the time of his arrest and his trial, he went to

Bellevue, the *deus ex machina*, and had his "diploma" endorsed. When the trial came off, it became necessary to subpœna the secretary of the college in order to prove that the endorsement appended to said document was genuine. This interesting foreigner was tried in two courts, at Special Sessions and at the Second District Police Court. At the former trial, the secretary of Bellevue Hospital Medical College testified that he had endorsed the document, believing the same to be a license, but he had no personal knowledge of its genuineness. At the second trial, the endorsement was admitted, but it was made, the secretary thought, upon the recommendation of Dr. Antonini, but as this gentleman was dead and the letter which was stated to have been written, endorsing Marini, had not been retained, this point was shrouded in mystery. Moreover, the singular statement was advanced that the signature was affixed, knowing that the instrument endorsed was a license and not a diploma. Now the law is explicit that diplomas may be endorsed, with certain qualifications, but licenses are not included, yet here admission is made that this man's paper, or whatever it may be called, was endorsed when the secretary knew that it was not a diploma.

Marini, on the first trial, was fined, but on the second, the presiding judge, a police justice, held that Marini was a legal practitioner, because his paper had been endorsed by the Bellevue Hospital Medical College, no matter what its former character had been. Supposing, therefore, that this ruling is correct, the Bellevue Hospital Medical College is directly responsible for legalizing a bogus document.

The second case is that of Siskind Treu, who is registered as follows: "Treu, Siskind, 16, Essex Board of Health, Cowne, Russia, 1862. Indorsed Bell, 1881." This turns out to be a nurse's certificate and not a diploma at all, so we are informed.

The third case is still more interesting, as it shows how good a doctor's endorsement of any given man may be. Within the past few weeks, New York City has been blest by the advent of two "experienced physicians," who, urged by a philanthropic yearning to benefit those unfortunates who may be suffering from "blood and skin diseases," have opened a shop for the sale of the wonderful remedies known as S. S. S. One of these humanitarians registered as a graduate of Jefferson Medical College of Philadelphia, 1847; the other, of the Savannah Medical College, 1856, and were promptly endorsed by Bellevue, upon the recommendation of Dr. Bozeman, of this city, so we are informed.

Is not this terrible condition of things enough to send a pang of sorrow through the heart of every true lover of the code of our fathers: to think that Bellevue, the school whose proud headline in its advertisement reads that, "The standard of medical ethics recognized by this college is embodied in the code of ethics of the American Medical Association," and which declares as its invariable rule, that "the Faculty will not grant a degree to any graduate of three or more years' standing, who does not exhibit to the secretary a certificate of membership in some medical society entitled to representation in the American Medical Association," should so far lapse from the paths of ethical rectitude as to endorse a paper which the secretary admitted, in evidence, he knew was not a diploma; to endorse a nurse's certificate as a diploma, and to endorse the diplomas of two advertising doctors who sell a proprietary medicine. Oh! this is too, too much. Well may the stanch advocates of the dear old code exclaim: "Ichabod, the glory is departed from Israel."

---

Office of the President,
American Public Health Association.
Washington, D. C., Jan. 31, 1884.

*To the Committee on Legislation, Erie Co. Medical Society:*

Gentlemen—I have read, with very great satisfaction, the copy of your proposed bill for the establishment of the Medical Faculty of the University of the State of New York, for the regulation of the practice of medicine in that State, and I heartily congratulate your committee upon the admirable and effective scheme you have devised, and especially upon that feature of it which entirely separates your proposed Faculty from schools and colleges. I regard it as of the first importance, indeed, as absolutely necessary, in any undertaking of this kind, that the licensing faculty should be wholly distinct and independent from that which teaches; and I hope to see the day when examinations will be conducted and degrees conferred by a board or faculty which has no pecuniary or other interest in the size of college classes. It is out of the question to expect that men who stand in the relation of instructors can be impartially critical of the merits of candidates.

I cordially wish you success in a cause in which I have myself made some effort. Your State should lead in the matter of medical reform, and I trust that it is now about to set an example that will be followed and imitated all over the Union, with the effect of ridding our profession of the burden of ignorance, illiteracy and incompetence, which now disgraces it.

Very truly yours,

ALBERT L. GIHON.

